# Outcome of allogeneic hematopoietic cell transplantation in patients with adult T‐cell leukemia

**DOI:** 10.1002/hon.2549

**Published:** 2018-09-10

**Authors:** Ayako Kamiunten, Masaaki Sekine, Takuro Kameda, Keiichi Akizuki, Yuki Tahira, Kotaro Shide, Haruko Shimoda, Koji Kato, Tomonori Hidaka, Yoko Kubuki, Kazuya Shimoda

**Affiliations:** ^1^ Department of Gastroenterology and Hematology, Faculty of Medicine University of Miyazaki Miyazaki Japan; ^2^ Department of Medicine and Biosystemic Science Kyusyu University Faculty of Medicine Fukuoka Japan

**Keywords:** adult T‐cell leukemia/lymphoma, allogeneic hematopoietic stem‐cell transplantation, chemotherapy

## Abstract

Adult T‐cell leukemia/lymphoma (ATL) is an aggressive peripheral T‐cell neoplasm, and the outcome of patients with ATL after chemotherapy is poor. Allogeneic hematopoietic stem‐cell transplantation (allo‐HSCT) is a curative treatment modality for ATL, and four factors, namely, age > 50 years, male recipient, lack of complete remission at transplantation, and transplantation of cord blood, were previously shown to be associated with poor survival. We retrospectively analyzed the outcome of 21 patients with ATL who had undergone allo‐HSCT at our hospital during a 3‐year period. Of 21 patients, all had at least one of the above risk factors, and 18 had two or more. With a median follow‐up of 19.7 months for living patients, the 1‐ and 2‐year overall survival (OS) rates after transplantation were 34% and 27%, respectively. All relapse/progression events occurred within 1 year after allo‐HSCT, and the cumulative incidence of relapse/progression at 1 year after allo‐HSCT was 46.9%. The 100‐day and 1‐year nonrelapse mortality (NRM) rates were 19% and 42%, respectively. No significant difference in OS was observed between myeloablative and reduced‐intensity conditioning regimens. The 3‐year OS (27%) of ATL patients who received allo‐HSCT and who had at least one adverse factor was somewhat poorer than the 3‐year OS of 33% identified in a nationwide study of allo‐HSCT in ATL patients in Japan. The high relapse/progression and NRM rates are major problems to be solved to achieve better outcome.

## 1 INTRODUCTION

Adult T‐cell leukemia/lymphoma (ATL) is an aggressive peripheral T‐cell lymphoma that is causally linked to infection with human T‐lymphotropic virus‐1 (HTLV‐1).^1^, ^2^, ^3^ According to the Shimoyama classification, ATL is classified into four types, namely, acute, lymphoma, chronic, and smoldering.^4^ The prognosis of acute‐ and lymphoma‐type ATL is poor when treated with chemotherapy.^5^ The 4‐year overall survival (OS) rates were 11% and 16% for acute and lymphoma types, respectively, and the OS plot did not reach a plateau.^6^ Allogeneic hematopoietic stem‐cell transplantation (allo‐HSCT) is considered to be a curative treatment option for patients with ATL.^7^ Nationwide retrospective studies in Japan demonstrated that the 3‐year OS rate was 33% in 386 ATL patients who underwent allo‐HSCT.^8^ Multivariate analysis revealed that four factors, specifically age > 50 years, male recipient, lack of complete remission (CR) at transplantation, and transplantation of cord blood, were associated with poor survival.

Allo‐HSCT is an effective treatment option, but not all ATL patients are appropriate candidates for this therapy. In previous studies, the median ages of acute‐ and lymphoma‐type ATL patients were 63 and 66 years, respectively, whereas the median age of patients who underwent allogeneic bone marrow transplantation (BMT) or peripheral blood stem‐cell transplantation (PBSCT) was 53 years, and that in patients who received cord blood transplantation (CBT) was 55 years.^6^, ^9^, ^10^ There has been no randomized comparison of chemotherapy alone vs allo‐HSCT. Kawada et al^11^ performed a retrospective analysis of treatment outcome in ATL patients and showed improved 3‐year OS in allo‐HSCT recipients over those receiving chemotherapy alone. In a retrospective nationwide study, Fuji et al^12^ also recently reported better OS in transplanted patients compared with nontransplanted patients. The objective of our study was to retrospectively analyze the outcome of ATL patients undergoing allo‐HSCT at our institution.

## 2 MATERIALS AND METHODS

### 2.1 Patients and data collection

Clinical data of 21 ATL patients who received allo‐HSCT from April 2014 to April 2017 at University of Miyazaki Hospital were retrospectively collected and reviewed.

This study was approved by the Research Ethics Committee of the Faculty of Medicine, University of Miyazaki.

### 2.2 Definitions and clinical outcome variables

Overall survival was calculated from the day of HSCT or diagnosis until death or last observation, as indicated. Patients who remained alive at the time of the last follow‐up were censored. The definition of a therapeutic response to chemotherapy was based on a previous report and its modification.^11^, ^13^ Response to treatment was divided into four categories: CR, partial remission (PR), stable disease (SD), and progressive disease (PD). Non‐CR was defined as PR + SD + PD. Myeloablative and reduced‐intensity conditioning regimens were defined according to the Center for International Blood and Marrow Transplant Research criteria.^14^ Nonrelapse mortality (NRM) was defined as death from any cause without disease relapse/progression.

### 2.3 Statistical analysis

The Kaplan‐Meier method was used to estimate OS, and the log‐rank test was used to compare OS between two groups. All data were analyzed using SPSS version 20 software (SPSS, Chicago, Illinois).

## 3 RESULTS

### 3.1 Patients and transplantation characteristics

From April 2014 to April 2017, 21 ATL patients received allo‐HSCT at our institute. The patient, disease, and transplant characteristics at HSCT are presented in Table 1. In brief, 12 patients (57%) were male, 19 (90.5%) were > 50‐year‐olds at transplantation, and the median age was 63 years (range, 40‐66). All 21 patients initially received induction chemotherapy such as VCAP‐AMP‐VECP (vincristine, cyclophosphamide, doxorubicin, and prednisone; doxorubicin, ranimustine, and prednisone; and vindesine, etoposide, carboplatin, and prednisone) or CHOP (cyclophosphamide, adriamycin, vincristine, and prednisone). Of 21 patients, two received mogamulizumab therapy; they received their final doses of mogamulizumab at 67 and 70 days before transplantation, respectively. The median time from diagnosis of ATL to transplantation was 147 days (53‐412), and the disease status at transplantation was CR in 1 case, PR in 10, SD in 2, and PD in 8. The PS at transplantation was 0 to 1 in all patients. Overall, 3, 3, 11, and 4 patients, respectively, had one, two, three, and four of the four reported factors that are associated with poor OS (age > 50 years, male recipient, lack of complete remission at transplantation, and transplantation of cord blood).

**Table 1:** Characteristics of patients with ATL after allogeneic hematopoietic stem cell transplantation

Characteristic	Allo‐HSCT patients (n = 21)
**Age**	
Year (range)	63 (40‐66)
**Sex**	
Male	12 (57%)
Female	9 (43%)
**Subtype of ATL**	
Acute	17 (81%)
Lymphoma	4(19%)
**Initial chemotherapy**	
CHOP or CHOP‐like regimen	16 (76.2%)
VCAP‐AMP‐VECP or VCAP‐AMP‐VECP‐like regimen	5 (23.8%)
**Mogamulizumab therapy**	2 (9.5%)
**Disease status at HSCT**	
CR	1 (4.8%)
PR	10 (47.6%)
SD	2 (9.5%)
PD	8 (38.1%)
**Performance status at HSCT**	
0	16 (76.2%)
1	5 (23.8%)
2	0 (0%)
3–4	0 (0%)
**Days from diagnosis to transplantation**	
Median (range)	147 (53–412)
**Source of stem cells**	
Bone marrow	11 (52.4%)
Peripheral blood	3 (14.3%)
Cord blood	7 (33.3%)
**HLA of stem cells**	
Matched	7 (33.3%)
One‐antigen mismatched	9 (42.9%)
Two‐antigen mismatched	4 (19%)
Three‐antigen mismatched	1 (4.8%)
**Conditioning regimen**	
MAC	7 (33.3%)
RIC	14 (66.7%)
**GVHD prophylaxis**	
Cyclosporine‐based	2 (9.4%)
Tacrolimus‐based	19 (90.6%)
MMF	7 (33.3%)

Abbreviations: ATL, adult T‐cell leukemia/lymphoma; CR, complete remission; HSCT, hematopoietic stem‐cell transplantation; MAC, myeloablative conditioning; MMF, mycophenolate mofetil; PD, progressive disease; PR, partial remission; RIC, reduced‐intensity conditioning; SD, stable disease.

Of 21 patients, 14 received reduced‐intensity conditioning that consisted of fludarabine with either busulfan or cyclophosphamide ± total body irradiation. The conditioning regimen was chosen by each attending physician. Donors were one antigen–mismatched unrelated in six cases, and cord blood in seven cases.

### 3.2 Outcomes

Primary graft failure was observed in one of six recipients of human leukocyte antigen (HLA)‐mismatched unrelated grafts and one of seven recipients of cord blood grafts, whereas the remaining 19 patients had evidence of initial engraftment. Of these 19 patients, the 15 who survived for 100 days after allo‐HSCT were assessed for acute graft‐versus‐host disease (aGVHD), which occurred at grades I, II, and III in 6, 4, and 1 patients, respectively. After HSCT, five of 10 patients whose initial response to chemotherapy was SD or PD achieved CR or PR.

**Figure 1:**
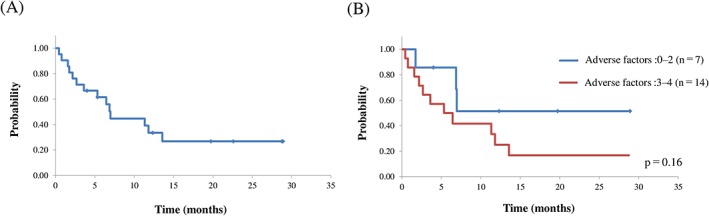
A, Overall survival from the time of transplantation; B, overall survival from the time of transplantation disaggregated by the number of adverse factors

Six patients died of disease relapse or progression, three died of infection, and five died of other causes. With a median follow‐up period of 19.7 months (range, 4.0‐28.9), the 1‐ and 2‐year OS rates of the whole group were 34% (95% confidence interval [CI], 12%‐55%) and 27% (95% CI, 6%‐48%), respectively. (Figure 1A). No difference was found in OS for myeloablative regimens versus reduced‐intensity stem cell transplantation (*P* = 0.75), or in OS disaggregated by the number of adverse factors. The 2‐year OS rate for patients with three or four adverse factors was 17%, and that for patients with two or fewer factors was 51% (*P* = 0.16) (Figure 1B).

**Figure 2:**
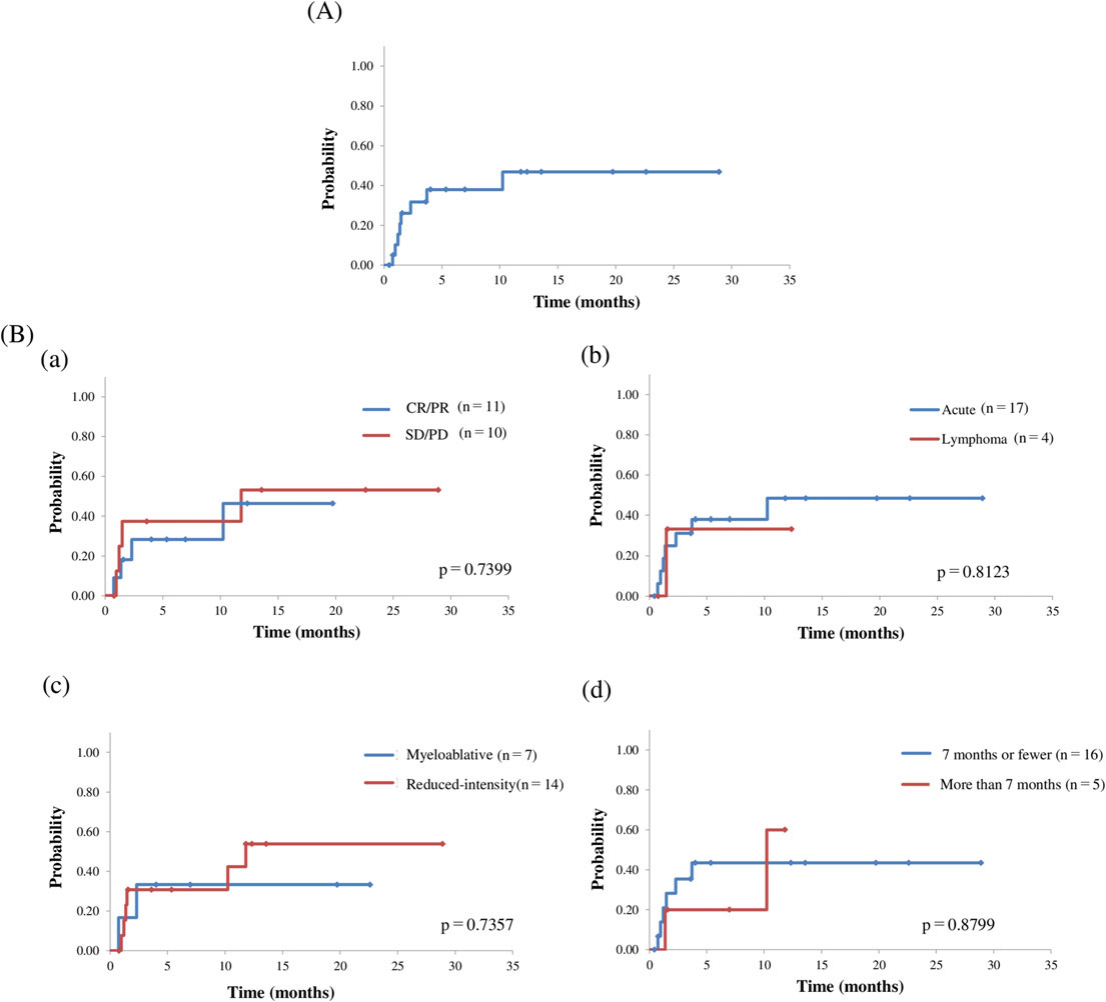
A, Cumulative incidence of relapse/progression; B, cumulative incidence of relapse/progression grouped according to risk factors. a, disease status at allo‐HSCT (CR/PR vs SD/PD); b, adult T‐cell leukemia/lymphoma clinical subtype (acute vs lymphoma type); c, conditioning regimen (myeloablative vs reduced‐intensity); d, time from diagnosis to transplantation (7 months or less vs more than 7 months). CR, complete remission; PD, progressive disease; PR, partial remission; SD, stable disease

During the observation period, eight of 21 patients (38.1%) relapsed or progressed at a median of 42.5 days (range, 22‐307 days) after allo‐HSCT, and all within 1 year. The cumulative incidence of relapse/progression at 2 years after allo‐HSCT was 46.9% (Figure 2A). Inoue et al^15^ reported that several factors were independently associated with risk of relapse/progression, namely, disease status of SD/PD at allo‐HSCT, lymphoma subtype, reduced‐intensity stem cell transplantation (RIST), and time from diagnosis to allo‐HSCT of more than 7 months; however, these risk factors had minimal predictive value for disease relapse/progression after allo‐HSCT in our cohort (Figure 2B). Among eight patients who suffered from ATL relapse/progression, one obtained CR and is still alive, one died of HHV6 encephalitis, and the remaining six died of relapse/progression.

**Figure 3:**
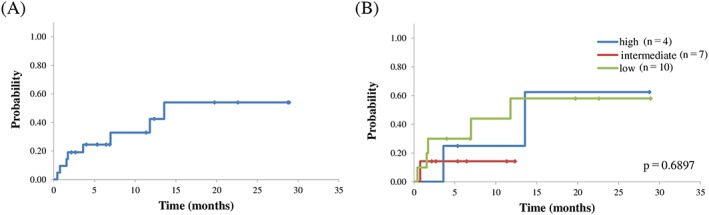
A, Nonrelapse mortality from the time of transplantation; B, nonrelapse mortality from the time of transplantation grouped according to ATL‐HCT‐PI

The cumulative incidences of NRM at 100 days and 1 year after transplantation were 19% (95% CI, 2%‐36%) and 42% (95% CI, 16%‐69%), respectively (Figure 3A). Recently, Yoshimitsu et al^16^ established ATL‐HCT‐PI, a predictive scoring system for NRM in allo‐HCT for ATL patients. ATL‐HCT‐PI consists of three factors: revised HCT‐CI, sex combination (female donor and male recipient), and age 64 years or older. ATL‐HCT‐PI could not categorize our ATL patients based on NRM, and the cumulative incidences of NRM at 1 year were 58%, 14.3%, and 25% in the low‐, intermediate‐, and high‐risk groups, respectively (*P* = 0.6897) (Figure 3B).

## 4 DISCUSSION

In this study, the 2‐year OS rate following allo‐HSCT was 27%. Comparatively, the 3‐year OS rate was 33% in a nationwide retrospective study of allo‐HSCT in 386 ATL patients in Japan.^8^ In that study, age > 50 years, male recipient, non‐CR disease status, and cord blood as the graft source were adversely associated with OS by multivariate analysis. As for the effect of graft source on OS, two other nationwide retrospective studies in Japan also suggested inferior OS rates for CBT compared with allo‐BMT or PBSCT; the 3‐year OS rate was 36% in 586 ATL patients receiving allo‐BMT or PBSCT, while the 2‐year OS rate was 20.6% in 175 ATL patients receiving CBT.^9^, ^10^ Both age > 50 years and non‐CR disease status at transplantation were independent risk factors in one of these reports,^9^ whereas neither age nor disease status at allo‐HSCT affected OS in the other report.^10^ In this study, 19 patients (90.5%) were older than 50 years, 12 (57%) were male recipients, 20 (95%) had non‐CR disease status at allo‐HSCT, and 7 (33%) underwent CBT. Every patient in our study had at least one adverse factor, and 18 (85.7%) had two or more. This might have led to the relatively poorer outcome of allo‐HSCT in ATL patients in this study compared with the nationwide study in Japan.

The high rate of relapse (38% at 1 year) and NRM (42% at 1 year) remains a significant problem, although some patients achieved longer survival. A retrospective study of allo‐HSCT in ATL patients showed that the development of grades 1 to 2 aGVHD was associated with improved OS compared with the absence of aGVHD.^10^, ^17^ In this study, 11 of 15 patients who survived for 100 days after allo‐HSCT developed grades 1 to 2 aGVHD, and its presence had little effect on their OS (mean survival time, 11.8 months in patients with aGVHD vs 6.9 months in those without aGVHD, *P* = 0.88). Inoue et al^15^ reported that four factors, namely, disease status of SD/PD at allo‐HSCT, lymphoma subtype, RIST, and time from diagnosis to allo‐HSCT more than 7 months, were independently associated with risk of relapse/progression. These risk factors, however, failed to classify our ATL patients into different disease relapse/progression risk categories after allo‐HSCT. As for pretransplant disease status, our patients in CR or PR had higher relapse/progression rates after allo‐HSCT compared with the report of Inoue et al,^15^ although the relapse/progression rates after allo‐HSCT were comparable in SD and PD patients. The smaller proportion of CR patients in this study (9% vs 67% in the report by Inoue et al) might be one reason why relapse/progression rates were higher in this study. The prognosis after relapse/progression in transplanted ATL was poor, and only one case (12.5%) obtained CR and was still alive at this writing.

Another reason for unfavorable OS after allo‐HSCT was higher NRM in our study. Yoshimitsu et al^16^ recently reported that three factors, namely, revised HCT‐CI, sex combination (female donor and male recipient), and age 64 years or older, were useful for predicting NRM in ATL patients after allo‐HCT (ATL‐HCT‐PI). In our study, even the low‐risk group defined by ATL‐HCT‐PI had a high NRM of 58%, with the following causes of death: disease relapse/progression (two cases), HHV‐6 encephalopathy, bacterial infection, thrombotic microangiopathy (TMA), and lymphoproliferative disorder (each one case). As for infection, additional two and one patients died of bacterial and fungal infections, respectively, in our overall sample, and infection was the most frequent cause of NRM (63%) in our study. Immunosuppressed patients with ATL might be at an increased risk of developing opportunistic infections.^18^

## 5 CONCLUSION

Allo‐HSCT results in long‐term survival in ATL patients in clinical practice, and some individuals may be cured. The use of induction chemotherapy to increase the CR rate before allo‐HSCT, as well as strategies to decrease NRM, might improve the outcome of allo‐HSCT in ATL patients.

## CONFLICT OF INTEREST

We have no financial relationships to disclose.

## FUNDING

This research was not supported by any specific grants, equipment, or drugs from funding agencies.
